# Combined Serum Albumin and Left Ventricular Ejection Fraction Predict All-Cause Death in Patients with Stable Coronary Artery Disease

**DOI:** 10.1155/2024/9969628

**Published:** 2024-03-31

**Authors:** Hua Zhang, Shaodong Qiu, Fei Chen, Xiaojun Wang

**Affiliations:** Department of Medical Ultrasound, The Second Affiliated Hospital of Guangzhou Medical University, No. 250, Changgang East Road, Haizhu District, Guangzhou, Guangdong, China

## Abstract

**Objective:**

To explore the feasibility of serum albumin (Alb) and left ventricular ejection fraction (LVEF) in predicting all-cause death (ACD) in patients with stable coronary artery disease (SCAD).

**Methods:**

Patients with SCAD were divided into 4 groups according to their Alb and LVEF levels: Group A: Alb ≤4 g/dL and LVEF > 50%; Group B: Alb ≤4 g/dL and LVEF ≤50%; Group C: Alb >4 g/dL and LVEF ≤50%; Group D: Alb >4 g/dL and LVEF >50%. The K–M curve and log-rank test were used to compare ACD among the four groups over three years. Receiver operating characteristic (ROC) curves were used to compare the efficacy of predicting ACD among the combination of Alb and LVEF and either Alb or LVEF alone. Cox regression analysis identified the influencing factors of ACD in patients with SCAD and detected the correlation between Alb and LVEF.

**Results:**

ACD occurred in 18 (8.9%) of 203 patients with SCAD, with an average follow-up of 26.53 ± 14.34 months. In the Kaplan‒Meier analysis, the risk of ACD in the four groups ranged from high to low: Group B (17.6%) > Group A (26.7%) > Group D (0.9%) > Group C (0%, *P* < 0.001). The ROC curve showed that the combination of Alb and LVEF (AUC = 0.888) had better predictive value for ACD than either Alb (AUC = 0.879) or LVEF alone (AUC = 0.651), *P* < 0.001. Multivariate Cox regression analysis showed that Alb ≤4 g/dL predicted ACD events after adjusting for baseline (HR: 12.16, 95% CI: 1.57 to 94.41; *P*=0.017) and treatment (HR: 19.36, 95% CI: 2.53–147.78, *P*=0.004). Alb was positively correlated with LVEF (*r* = 0.22, *P*=0.002).

**Conclusions:**

Alb combined with LVEF is more effective than a single index in predicting ACD in SCAD and could be used as a new model to judge the prognosis of SCAD.

## 1. Introduction

Stable coronary heart disease (SCAD) refers to the presence of atherosclerotic plaques in the coronary arteries or is accompanied by uncomfortable symptoms of angina pectoris, and the stable period after the onset of acute coronary syndrome (ACS) also belongs to this category [1, 2]. SCAD is one of the most common types of coronary heart disease, and the incidence rate over 60 years may exceed 12%, making this a major public health concern [3]. SCAD can induce heart failure, arrhythmia, sudden death, stroke, and other adverse complications, which seriously threaten the health of the global population.

C-reactive protein (CRP) and high-sensitivity cardiac troponin T (hs-cTnT) are classic indicators commonly used to evaluate the prognosis of SCAD [4, 5]. Serum albumin (Alb) can not only maintain the plasma colloid osmotic pressure and reduce the extravasation of liquid into the intercellular matrix but also can be anti-inflammatory and can be an antioxidant and plays an important role in acute and chronic heart failure (HF), acute coronary syndrome (ACS), septic shock, nephrotic syndrome, cirrhosis, stroke, and malignant tumours [6–12]. In recent years, studies have shown that Alb is closely related to the prognosis of SACD after percutaneous coronary intervention [13, 14]. Left ventricular ejection fraction (LVEF) is an important index to measure left ventricular systolic function and guide medical treatment of HF [15]. It is also an important indicator for evaluating the prognosis of coronary heart disease [16, 17]. At present, there was no report about the prognosis of SCAD by combined Alb and LVEF. Therefore, our aim is to explore the application value of Alb combined with LVEF in evaluating the all-cause mortality risk of SCAD patients through this retrospective study.

## 2. Methods

### 2.1. Data Download

This was a single-centre retrospective study. As a secondary analysis, all the data were derived from the published article shared by Suzuki et al. [13]. We obtained the data from the “DATADRYAD” database (https://datadryad.org/stash/). Dryad is a public database, and users can download data freely. Dryad data package: Suzuki, et al. Data from: prognostic significance of serum albumin in patients with stable coronary artery disease treated by percutaneous coronary intervention, Dryad, Dataset, https://doi.org/10.5061/dryad.fn6730j [18]. A total of 204 patients were newly diagnosed with SCAD, were admitted to Shinonoi General Hospital, Japan, from October 2014 to October 2017, and agreed to receive percutaneous coronary intervention (PCI) treatment. Patients with malignant tumours or old myocardial infarctions were excluded. The study was approved by the Shinonoi General Hospital Ethics Committee, and all patients signed informed consent forms. The data collected included clinical characteristics, medical history, major risk factors, medications, laboratory, echocardiography, and angiography results, and comorbidities during follow-up.

### 2.2. Define Grouping and Prognosis

**Figure 1:**
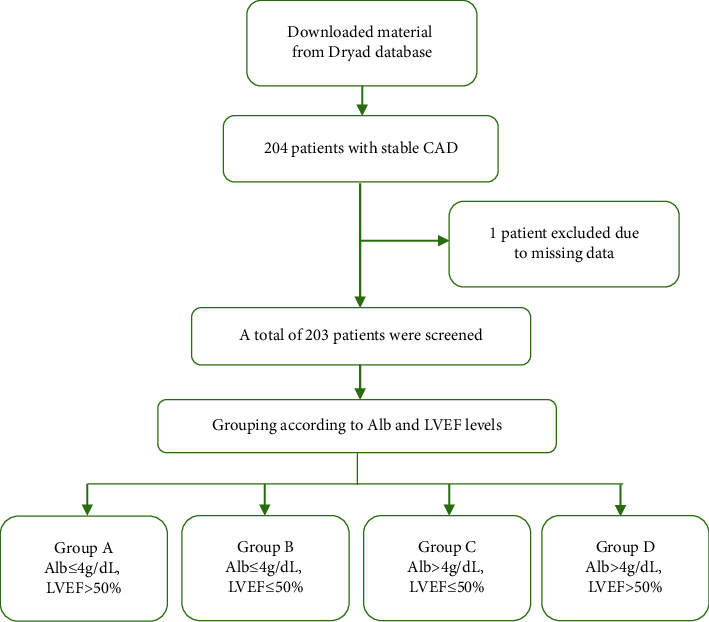
Flowchart of this study.

SCAD was defined as coronary angiography showing ≥90% epicardial coronary artery stenosis or ≥75% coronary artery stenosis with angina symptoms induced by drugs or exercise stress tests. Alb was detected by the bromocresol purple assay and the LABOSPECT 008 analyser (Hitachi Ltd., Tokyo, Japan) at admission. The normal value range of Alb for adults is 4–6 g/dL. According to the cut-off point of 4 g/dL, all patients were divided into a hypoalbuminemia group (Alb ≤4 g/dL) and a normal albuminemia group (Alb >4 g/dL). The left ventricular systolic function was obtained by measuring LVEF in the Teich method with echocardiography. The LVEF equals the LV stroke volume (LVSV) divided by the LV end-diastolic volume (LVEDV) and is usually expressed as a percentage. According to the HF guidelines [15], we regarded patients with LVEF ≤50% as the group with reduced cardiac function and patients with LVEF >50% as the group with normal cardiac function. Then, all patients were divided into four groups: Group A: Alb ≤4 g/dL and LVEF >50%; Group B: Alb ≤4 g/dL and LVEF ≤50%; Group C: Alb >4 g/dL and LVEF ≤ 50%; and Group D: Alb >4 g/dL and LVEF >50% (Figure 1).

All patients were followed up until the occurrence of ACD. Survival time was calculated from the date of PCI until an adverse event occurred.

### 2.3. Statistical Analysis

Continuous data are presented as the mean ± standard deviation (SD), whereas categorical data are presented as percentages (%). One-way analysis of variance (ANOVA) was used to compare continuous variables among groups, and the chi-square or Fisher's exact test was used for categorical variables. To compare the survival differences among the four groups composed of different Alb and LVEF, Kaplan–Meier (K–M) curves were plotted, and the log-rank test was used for statistical analysis. Receiver operating characteristic (ROC) curves were used to compare the discriminative power among the combination of Alb and LVEF and either Alb or LVEF alone. Then, the Youden index was calculated using the formula sensitivity + specificity −1. In the univariate analysis of ACD in patients with SCAD, all variables were analysed by the log-rank test, and variables with *P* < 0.10 were screened. Continuous variables were classified according to the mean value. Multivariate Cox regression was stratified according to the baseline, treatment, and laboratory data. Finally, correlation analysis and scatter plots were used to detect the relationship between Alb and LVEF. All statistical tests were two tailed, and *P* < 0.05 was considered statistically significant, with the tests being performed using SPSS version 27.0.1 and MedCalc version 20.116.

## 3. Results

### 3.1. Clinical Characteristics of the Four Groups

After excluding one patient whose LVEF data were missing, 203 patients with SCAD were included in the study. All patients were divided into four groups depending on their Alb and LVEF. In Table 1, those in the four groups were on average 72.6 ± 10.4 years old. Males accounted for 69.5% (141/203). The mean body mass index (BMI) was 23.6 ± 0.5.

Group C had the highest proportion of smoking (83.3%), whereas Group D had the highest percentage of dyslipidaemia (63%). The lowest mean LVEF was 34.5 ± 7.5% in Group C, followed by 40.4 ± 6.5% in Group B. There were no differences in blood pressure (BP) or additional previous medical history.

The average statin use rate of the four groups exceeded 50%. Groups B and C belonged to the heart failure group and antiheart failure drugs were used more commonly than in the other two groups.

Coronary angiography showed that 50.2% of the patients had bifurcation lesions, only 5% had chronic obstruction and left main artery lesions, and the highest degree of coronary artery calcification in Group A was 25.7%. A total of 94.6% of the patients were implanted with drug-eluting stents (DES). Nearly 1/4 of the patients underwent multivessel percutaneous coronary intervention (PCI).

The average Alb, hemoglobin (Hb), and low-density lipoprotein cholesterol (LDL-C) of Groups A and B were lower than those of the other two groups, and the lowest average Alb of Group B was only 3.3 ± 0.4 g/dL. All four groups had an estimated glomerular filtration rate (eGFR) less than 90 mL/min/1.73 m^2^, and the lowest level in Group B was 45.7 ± 23.5 mL/min/1.73 m^2^. The levels of total cholesterol (CHOL) and triglycerides (TG) in Groups C and D were the highest, and the level of C-reactive protein (CRP) in Group B was higher than those in the other three groups (*P* < 0.001).

### 3.2. Survival Analysis of ACD

**Figure 2:**
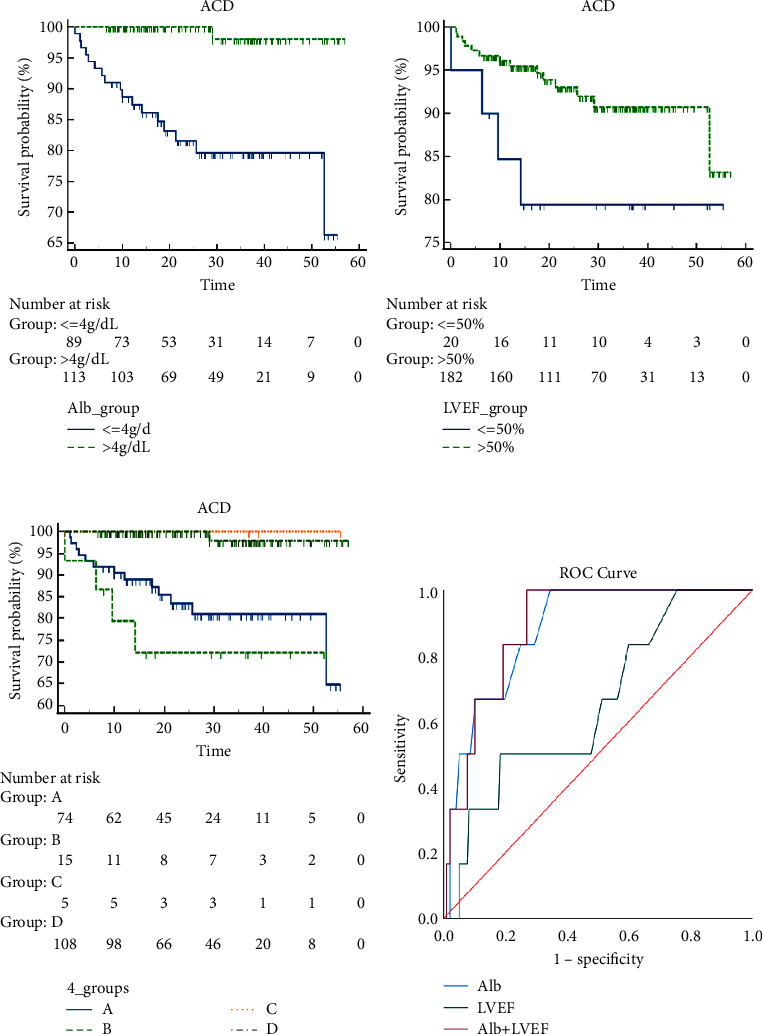
K–M curves for ACD based on Alb (a), or LVEF (b), or the combination of Alb and LVEF (c). ROC curve for the combination of Alb and LVEF, and either Alb or LVEF alone (d). K–M: Kaplan–Meier; ACD: all cause death; ROC: receiver operating characteristic.

In the average follow-up period of 26.53 ± 14.34 months, 18/203 (8.9%) SCAD patients developed an ACD. As shown in Figure 2, the risk of ACD with Alb ≤4 g/dL was significantly higher than that with Alb >4 g/dL (Alb ≤4 g/dL group: 19.1% (17/89) vs. Alb >4 g/dL group: 0.9% (1/114), *P* < 0.001). LVEF >50% reduced the risk of ACD, but the difference was not significant (LVEF >50% group: 7.7% (14/182) vs. LVEF ≤50% group: 19% (4/21), *P* = 0.095). Based on the K–M analysis, the risk of ACD in the four groups ranged from high to low: Group B (17.6% (13/74)) > Group A (26.7% (4/15)) > Group D (0.9% (1/108)) > Group C (0% (0/6), *P* < 0.001).

According to the ROC curve, combined Alb and LVEF (AUC = 0.888) had a better predictive value for ACD than Alb (AUC = 0.879) or LVEF (AUC = 0.651) alone, with a Youden index = 0.680, *P* < 0.001 (Figure 2(d)).

### 3.3. Cox Regression for ACD

A univariate analysis identified the following diagnostic risk factors for ACD: age, BMI, past smoker, DLP, LVEF, aspirin, statin, ARB, stent, Alb, eGFR, ALT, CHOL, HDL-C, LDL-C, and HbA1c (*P* < 0.10, Table 2). Alb combined with LVEF was subjected to hierarchical multivariate Cox regression analysis based on baseline, treatment, and laboratory data to predict the risk of ACD (Table 3). Alb ≤4 g/dL predicted ACD events after adjusting for baseline (hazard ratio (HR): 12.16, 95% confidence interval (CI): 1.57 to 94.41; *P*=0.017) and treatment (HR: 19.36, 95% CI: 2.53–147.78, *P*=0.004). No significant differences were found between Alb in laboratory stratification and LVEF in ACD risk prediction (*P* > 0.05).

### 3.4. Correlation between Alb and LVEF

**Figure 3:**
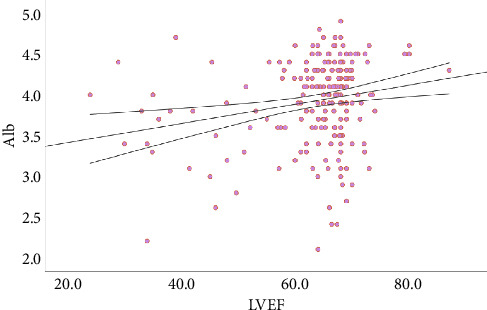
Scatter plots showed the correlation between Alb and LVEF.

As shown in Figure 3, scatter plots were generated, and Pearson's correlation coefficients were calculated to explore correlations between Alb and LVEF. There was a significant positive correlation between Alb levels and LVEF (*r* = 0.22, *P* = 0.002).

## 4. Discussion

This study mainly retrospectively analysed 203 newly diagnosed SCAD patients, compared the difference in ACD under different combinations of Alb and LVEF, and discussed whether the predictive efficacy of Alb and LVEF in evaluating ACD was different from that of Alb or LVEF alone.

Alb is a single-chain protein composed of 580–585 amino acids, accounting for 60% of the total plasma protein [19]. It is only generated by the liver and participates in various pathological and physiological functions [20]. The normal human serum Alb concentration range is 4–6 g/dL. Usually, less than 4 g/dL can be considered a decrease in albumin concentration [21], and some studies have considered that less than 3.5 g/dL as hypoalbuminemia [10, 22, 23]. There is a prevalence of 21–70% of hypoalbuminemia in hospitalized individuals among acutely ill patients [24]. Hypoalbuminemia has been linked to morbidity and mortality in hospitalized patients [25], including those with coronavirus disease 2019 (COVID-19), which is associated with a higher incidence of cardiac injury and other organ damage [26]. A study of 8,750 patients with acute myocardial infarction found that admission Alb levels were an independent predictor of long-term ACD [27]. There is evidence that hypoalbuminemia has been associated with reduced survival in patients with acute and chronic HF and ACS [10, 12, 28]. Moreover, the predictive value of hypoproteinaemia is related not only to the absolute value of albumin but also to the degree of albumin decline. A greater decrease in Alb in 5449 patients undergoing PCI is associated with a higher risk of long-term major adverse cardiac events [29].

Our study showed that the survival time of SCAD patients with Alb ≤4 g/dL was significantly lower than that of SCAD patients with Alb >4 g/dL. According to the multivariate Cox regression, the predicted risk of ACD in SCAD patients with Alb ≤4 g/dL at baseline and after PCI treatment was 12.16 and 19.36 times higher than that in SCAD patients with Alb >4 g/dL, respectively. Alb ≤4 g/dL was an independent predictor of ACD in SCAD patients. We speculated that the mechanism of hypoalbuminemia in SCAD may be related to various factors. Hypoalbuminemia could lead to fluid extravasation into the interstitial space, which causes pulmonary oedema and fluid retention and aggravates myocardial dysfunction by promoting myocardial oedema [30, 31]. Hypoalbuminemia can increase blood viscosity, increase the risk of vascular thrombosis [32, 33], and may also accelerate the damage of inflammation and oxidative stress on endothelial function [34].

HF is a common adverse complication of SCAD and may be associated with DNA-dependent protein kinase catalytic subunits (DNA-PKcs) [35]. LVEF is commonly used to measure left ventricular systolic function as an important tool to evaluate the prognosis of cardiovascular diseases such as CAD, cardiomyopathy, and valvular heart disease and has been used to guide the treatment of HF [15]. Both LVEF and scarring were independent risk factors for all-cause and cardiac death [36]. Our study showed that the survival rate of the K–M curve consistent with SCAD decreased with decreasing LVEF, suggesting that LVEF was associated with the prognosis of patients with SCAD.

When establishing the ROC curve of ACD (Figure 2(d)), the results show that the optimal cut-off values of Alb and LVEF are 3.8 g/dL and 68%, respectively. However, we did not use this result as the basis for grouping, considering that it is related to the basic conditions of the patients during the study enrolment period and the relevant parameters of the ROC curve model. Therefore, the clinically recognized cut-off value Alb of 4.0 g/dL and LVEF of 50% was finally selected as the cut-off with the patients divided into four groups for statistical analysis. The serum Alb level was determined by laboratory examination, while LVEF was determined by imaging index. We wanted to explore whether the combination of Alb and LVEF could bring new findings to the ACD assessment of SCAD patients. At present, there is no report on the ACD risk assessment of SCAD patients by combining Alb and LVEF, so it was an innovation of this study. We used the ROC curve to evaluate the ACD of SCAD in three cases of either Alb or LVEF alone and combined Alb and LVEF. The results showed that the test efficiency of the combined index was significantly higher than that of the single index, suggesting that the combined indicator may be a new important reference indicator for evaluating the prognosis of SCAD.

### 4.1. Limitations

There were still some deficiencies in this study. First, due to the limited research time, the number of patients in Group B and Group C was relatively small, which easily caused deviations in the statistical results. Second, only Alb was included after the adjustment of the baseline and treatment variables by the Cox regression equation, but not LVEF. Third, the correlation analysis between Alb and LVEF only suggested a weak correlation (*r* = 0.22), which did not rule out the correlation with the screening of LVEF cut-off values or the small number of enrolled patients. Further research was conducted to expand the sample size and try to conduct statistical analysis on different cut-off values of LVEF to observe whether there were different outcomes.

## 5. Conclusion

Serum Alb concentration is associated with many diseases and is often used as an important evaluation indicator for chronic liver disease, kidney disease, malignant tumours, and other diseases. In recent years, it has been found that Alb is also of great significance for the cardiovascular system, especially in patients with SCAD. Our study combined Alb and LVEF to evaluate the risk of ACD in SCAD patients, indicating that the dual-index evaluation has advantages over the single-index evaluation, and Alb was an independent risk factor affecting the prognosis of ACD. With the continuous deepening of research and the accumulation of sample size, there will be stronger evidence to support and provide a new idea for accurately judging the prognosis of SCAD patients.

## Figures and Tables

**Table 1:** Clinical characteristics of four groups of patients with SCAD.

	Total (*n* = 203)	Group A (*n* = 74)	Group B (*n* = 15)	Group C (*n* = 6)	Group D (*n* = 108)	*P* value
*Baseline*						
Age (years)	72.6 ± 10.4	76.0 ± 10.9	75.7 ± 10.9	60.2 ± 7.1	70.1 ± 9.3	<0.001
Sex (male)	141 (69.5)	50 (67.6)	9 (60.0)	6 (100.0)	76 (70.4)	0.162
BMI	23.6 ± 0.5	22.8 ± 4.3	20.8 ± 3.4	23.1 ± 3.5	24.5 ± 3.5	<0.001
SBP (mmHg)	136.4 ± 20.5	132.3 ± 20.8	143.5 ± 32.9	127.7 ± 20.8	138.6 ± 17.5	0.735
DBP (mmHg)	77.5 ± 13.2	75.3 ± 14.3	80.0 ± 19.1	78.7 ± 5.4	78.5 ± 11.7	0.478
Past smoker (%)	100 (49.3)	30 (40.5)	4 (26.7)	5 (83.3)	61 (56.5)	0.013
Hypertension (%)	150 (73.9)	48 (64.9)	10 (66.7)	5 (83.3)	87 (80.6)	0.099
DM (%)	72 (35.5)	28 (37.8)	5 (33.3)	2 (33.3)	37 (34.3)	0.962
DLP (%)	103 (50.7)	25 (33.8)	7 (46.7)	3 (50.0)	68 (63.0)	0.002
OCI (%)	35 (17.2)	14 (18.9)	5 (33.3)	1 (16.7)	15 (13.9)	0.346
PAD (%)	53 (26.1)	23 (31.1)	4 (26.7)	1 (16.7)	25 (23.1)	0.632
AF (%)	26 (12.8)	11 (14.9)	3 (20.0)	1 (16.7)	11 (10.2)	0.646
LVEF (%)	63.3 ± 9.8	65.3 ± 4.8	40.4 ± 6.5	34.5 ± 7.5	66.6 ± 4.8	<0.001

*Medication*						
Aspirin (%)	201 (99.0)	73 (98.6)	14 (93.3)	6 (100.0)	108 (100.0)	0.211
Thienopyridines (%)	199 (98)	73 (98.6)	73 (98.6)	6 (100.0)	106 (98.1)	0.688
Warfarin (%)	5 (2.5)	1 (1.4)	1 (6.7)	0 (0.0)	3 (2.8)	0.670
DOAC (%)	21 (10.3)	10 (13.5)	1 (6.7)	1 (16.7)	9 (8.3)	0.635
Ezetimibe (%)	3 (1.5)	1 (1.4)	0 (0.0)	0 (0.0)	2 (1.9)	0.866
PPI (%)	133 (65.5)	47 (63.5)	10 (66.7)	4 (66.7)	72 (66.7)	0.977
Statins (%)	110 (54.2)	30 (40.5)	9 (60.0)	3 (50.0)	68 (63.0)	0.027
ACEI (%)	19 (9.4)	4 (5.4)	5 (33.3)	0 (0.0)	10 (9.3)	0.025
ARB (%)	88 (43.3)	31 (41.9)	9 (60.0)	2 (33.3)	46 (42.6)	0.569
*β*-blocker (%)	55 (27.1)	17 (23.0)	8 (53.3)	5 (83.8)	25 (23.1)	0.002
MRA (%)	101 (49.8)	2 (2.7)	4 (26.7)	2 (33.3)	3 (2.8)	0.002

*Coronary intervention*						
LMT (%)	13 (6.4)	8 (10.8)	0 (0.0)	1 (16.7)	4 (3.7)	0.099
Calcification (%)	29 (14.3)	19 (25.7)	1 (6.7)	1 (16.7)	8 (7.4)	0.006
Bifurcation (%)	102 (50.2)	38 (51.4)	7 (46.7)	5 (83.3)	53 (48.1)	0.365
CTO (%)	12 (5.9)	3 (4.1)	0 (0.0)	0 (0.0)	9 (8.3)	0.252
Stent BMS (%)	11 (5.4)	4 (5.4)	2 (13.3)	0 (0.0)	5 (4.6)	0.754
DES (%)	192 (94.6)	70 (94.6)	13 (86.7)	6 (100.0)	103 (95.4)	0.545
Multivessel PCI (%)	53 (26.1)	24 (32.4)	2 (13.3)	3 (50.0)	24 (22.2)	0.148

*Laboratory data*						
Alb (g/dL)	3.9 ± 0.5	3.5 ± 0.4	3.3 ± 0.5	4.3 ± 0.3	4.3 ± 0.2	<0.001
Hb (g/dL)	13.6 ± 2.0	12.4 ± 2.0	12.4 ± 2.1	15.0 ± 0.9	13.6 ± 2.0	<0.001
eGFR (mL/min/1.73 m^2^)	60.9 ± 24.3	53.3 ± 29.3	45.7 ± 23.5	52.5 ± 15.8	68.9 ± 24.2	<0.001
AST (U/L)	24.7 ± 10.9	24.5 ± 12.2	24.3 ± 14.3	23.0 ± 4.5	24.9 ± 11.0	0.973
ALT (U/L)	20.9 ± 12.2	18.3 ± 11.9	19.5 ± 15.8	21.2 ± 11.9	22.8 ± 12.2	0.101
CHOL (mg/dL)	185.4 ± 35.8	171.6 ± 30.3	174.0 ± 39.7	208.7 ± 30.4	195.4 ± 35.8	<0.001
TG (mg/dL)	134.5 ± 98.2	117.6 ± 94.2	93.7 ± 44.3	119.3 ± 38.9	152.8 ± 105.3	0.036
HDL-C (mg/dL)	50.1 ± 13.2	48.1 ± 12.3	45.0 ± 11.8	57.5 ± 29.7	51.8 ± 12.4	0.062
LDL-C (mg/dL)	110.0 ± 28.6	99.1 ± 25.3	105.9 ± 38.0	129.0 ± 17.4	117.0 ± 27.5	<0.001
CRP (mg/dL)	0.5 ± 1.1	0.8 ± 1.4	1.1 ± 1.8	0.2 ± 0.3	0.5 ± 1.1	<0.001
HbA1c (%)	7.3 ± 13.6	6.3 ± 1.3	6.7 ± 1.5	6.7 ± 0.9	6.3 ± 0.8	0.608

BMI: body mass index; SBP: systolic blood pressure; DBP: diastolic blood pressure; DM: diabetes mellitus; DLP: dyslipidemia; OCI: old cerebral infarction; PAD: peripheral artery disease; AF: atrial fibrillation; LVEF: left ventricular function; DOAC: direct oral anticoagulants; PPI: proton pump inhibitor; ACEI: angiotensin converting enzyme inhibitor; ARB: angiotensin receptor blocker; MRA: mineralocorticoid receptor antagonist; LMT: left main trunk; CTO: chronic total occlusion; BMS: bare mental stent; DES: drug eluting stents; PCI: percutaneous coronary intervention; Alb: albumin; Hb: hemoglobin; eGFR: estimated glomerular filtration rate; AST: aspartate transaminase; ALT: alanine aminotransferase; CHOL: cholesterol; TG: triglyceride; HDL-C: high-density lipoprotein cholesterol; LDL-C: low-density lipoprotein cholesterol; CRP: C-reactive protein.

**Table 2:** Univariate analysis for ACD.

Variables	HR	95% CI	*P* value
*Baseline*			
Age (≤72 vs. >72 y)	3.82	1.51–9.64	0.005
Sex (male vs. female)	1.00	0.37–2.68	0.994
BMI (≤23.6 vs. >23.6)	0.21	0.08–0.53	0.001
SBP (≤136 vs. >136 mmHg)	0.60	0.24–1.53	0.291
DBP (≤78 vs. >78 mmHg)	1.10	0.43–2.78	0.837
Past smoker (no vs. yes)	0.33	0.13–0.84	0.021
Hypertension (no vs. yes)	0.86	0.29–2.51	0.789
DM (no vs. yes)	0.51	0.20–1.35	0.180
DLP (no vs. yes)	0.19	0.08–0.49	<0.001
OCI (no vs. yes)	1.43	0.42–4.89	0.565
PAD (no vs. yes)	2.55	0.89–7.29	0.081
AF (no vs. yes)	2.70	0.64–11.36	0.173
LVEF (≤50 vs. >50%)	3.64	0.79–16.71	0.096

*Medication*			
Aspirin (no vs. yes)	0.0	0.0–0.11	0.007
Thienopyridines (no vs. yes)	0.23	0.01–5.15	0.359
Warfarin (no vs. yes)	6.14	0.23–166.92	0.281
DOAC (no vs. yes)	1.09	0.24–5.03	0.908
Ezetimibe (no vs. yes)	—	—	0.551
PPI (no vs. yes)	0.73	0.28–1.91	0.524
Statins (no vs. yes)	0.35	0.14–0.90	0.030
ACEI (no vs. yes)	0.96	0.22–4.23	0.964
ARB (no vs. yes)	2.21	0.87–5.65	0.098
*β*-blocker (no vs. yes)	1.09	0.38–3.12	0.876
MRA (no vs. yes)	3.53	0.43–29.04	0.242

*Coronary intervention*			
LMT (no vs. yes)	1.60	0.28–9.05	0.594
Calcification (no vs. yes)	2.24	0.57–8.82	0.250
Bifurcation (no vs. yes)	1.47	0.58–3.71	0.417
CTO (no vs. yes)	—	—	0.325
Stent (BMS vs. DES)	0.09	0.01–0.57	0.010
Multivessel PCI (no vs. yes)	1.08	0.38–3.09	0.886

*Laboratory data*			
Alb (≤4 vs. >4 g/dL)	0.12	0.05–0.31	<0.001
Hb (≤13.6 vs. >13.6 g/dL)	0.61	0.24–1.55	0.301
eGFR (≤60.9 vs. >60.9 mL/min/1.73 m^2^)	0.33	0.13–0.85	0.022
AST (≤24.7 vs. >24.7 U/L)	0.75	0.29–1.94	0.556
ALT (≤20.9 vs. >20.9 U/L)	0.32	0.12–0.83	0.020
CHOL (≤185.4 vs. >185.4 mg/dL)	0.25	0.09–0.72	0.010
TG (≤134.5 vs. >134.5 mg/dL)	0.64	0.25–1.65	0.356
HDL-C (≤50.1 vs. >50.1 mg/dL)	0.41	0.16–1.03	0.057
LDL-C (≤110.0 vs. >110.0 mg/dL)	0.34	0.14–0.87	0.024
CRP (≤0.5 vs. >0.5 mg/dL)	0.50	0.12–2.01	0.328
HbA1c (≤7.3 vs. >7.3%)	10.65	3.10–36.62	<0.001

**Table 3:** Multivariable Cox regression for ACD.

Variables	HR	95% CI	*P* value
Adjusted for baseline			
Age, BMI, past smoker, DLP, PAD			
Alb	12.16	1.57–94.41	0.017
LVEF	0.54	0.17–1.69	0.287
Adjusted for treatment			
Aspirin, stains, ARB, stent			
Alb	19.36	2.53–147.48	0.004
LVEF	0.84	0.25–2.78	0.769
Adjusted for laboratory			
eGFR, ALT, CHOL, HDL-C, LDL-C, HbA1c			
Alb	2.47*E* + 6	0.0–3.45*E* + 116	0.924
LVEF	0.75	0.08–6.78	0.799

## Data Availability

We obtained the data from the “DATADRYAD” database (https://datadryad.org/stash/). Dryad was a public database, and users could download data freely. Dryad data package: Suzuki, et al. Data from: prognostic significance of serum albumin in patients with stable coronary artery disease treated by percutaneous coronary intervention, Dryad, Dataset, https://doi.org/10.5061/dryad.fn6730j.
